# Effectiveness and Patient Experiences of Rhenium Skin Cancer Therapy for Nonmelanoma Skin Cancer: Interim Results from the EPIC-Skin Study

**DOI:** 10.2967/jnumed.124.267988

**Published:** 2024-09

**Authors:** Siddhartha Baxi, Saima Vohra, Angela Hong, Nicola Mulholland, Martin Heuschkel, Gerhard Dahlhoff, Giuseppe Cardaci, Siroos Mirzaei, Mike Sathekge

**Affiliations:** 1 Genesis Cancer Care, John Flynn Hospital, Tugun, Queensland, Australia;; 2 Avion Medical Skin Centres, North Melbourne, Victoria, Australia;; 3 Genesis Cancer Care, North Shore Health Hub, St. Leonards, New South Wales, Australia;; 4 Melanoma Institute Australia, Poche Centre, Crows Nest, New South Wales, Australia;; 5 Department of Nuclear Medicine, King’s College Hospital NHS Foundation Trust, London, United Kingdom;; 6 Department of Nuclear Medicine, University Medical Center Rostock, Rostock, Germany;; 7 OncoBeta International GmbH, Munich, Germany;; 8 Department of Nuclear Imaging, Hollywood Private Hospital, Nedlands, Western Australia, Australia;; 9 Clinic Ottakring, Institute of Nuclear Medicine with PET Center, Vienna, Austria; and; 10 Department of Nuclear Medicine, Steve Biko Academic Hospital, Pretoria, South Africa

**Keywords:** nonmelanoma skin cancer, Rhenium SCT, radionuclide

## Abstract

Nonmelanoma skin cancer and its treatment represent a significant global cancer burden for health care systems and patients. Rhenium skin cancer therapy (Rhenium SCT) is a novel noninvasive radionuclide nonmelanoma skin cancer treatment, which can be provided in a single outpatient session. The aim of this prospective, multicenter, single-arm, international, phase IV study (EPIC-Skin) is to assess clinic- and patient-reported outcomes of Rhenium SCT as a treatment for basal cell carcinoma (BCC) and squamous cell carcinoma (SCC). **Methods:** Eligible patients had biopsy-proven stage I or stage II BCC or SCC lesions no more than 3 mm deep and no larger than 8 cm^2^ in area. Rhenium SCT resin was applied to an adhesive foil affixed to the target lesion in a single session. Interim efficacy and safety analysis were planned once 50% of target patients had recorded a 6-mo follow-up visit. Primary outcome is the proportion of lesions achieving complete response using modified RECIST. Secondary and other outcome measures include patient-reported quality of life (QoL), treatment comfort, and cosmesis. **Results:** A total of 182 patients was enrolled and administered Rhenium SCT (50 Gy dose to deepest point of target) to at least 1 BCC or SCC. Of 81 patients who reached the 6-mo posttreatment follow-up, it was found that 97.2% (103/106) of lesions showed complete responses and 2.8% (3/106) had partial responses. Improvements in QoL were also reported, whereas no patients reported any pain or discomfort during treatment. Adverse events were reported in 15.9% (29/182) of patients and were rated grade 1 (*n* = 19), grade 2 (*n* = 9), or grade 3 (*n* = 1). **Conclusion:** This preliminary analysis of the EPIC-Skin study indicates that Rhenium SCT is safe and effective for the treatment of BCC and SCC and is associated with significant QoL improvements. It will be particularly beneficial for lesions that are difficult to treat surgically because of size and location. It is also beneficial for patients with comorbidities or those unable to receive conventional fractionated radiotherapy.

## Visual Abstract

**Figure:**
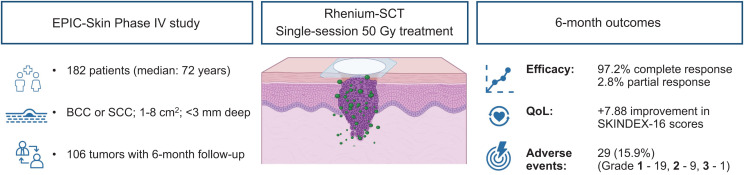

Nonmelanoma skin cancer (NMSC), including basal cell carcinoma (BCC) and cutaneous squamous cell carcinoma (SCC), is the most common form of skin cancer, with the highest incidence seen in Australia (incidence > 1,000/100,000 person years for BCC) (1–3). New cases and deaths from NMSC are predicted to increase by at least 1.5 times by 2044 (4). Most lesions develop on ultraviolet light–exposed skin, such as the head and neck and upper torso regions, resulting in a considerable impact on quality of life (QoL) (2*,*^5^). Other predisposing factors include age, sex (affecting mostly men), genetics, and immunosuppression (4*,*^6^). Prior diagnoses also substantially increase the risk of new lesions, which can lead to significant disease and treatment burden, impacting QoL (2*,*^5^).

Several definitive treatment options are available including surgery, cryotherapy, topicals (such as 5-fluorouracil and imiquimod), and conventional external beam radiotherapy (7). Treatments including local excision, plastics procedures, and Mohs surgery report the highest control rates for NMSCs of 94%–97% (8). Surgical challenges include patient selection from a technical or medical fitness point of view, particularly in elderly patients (9), and fatigue (10).

External beam radiotherapy, delivered in a fractionated or hypofractionated protocol, using surface brachytherapy solutions, superficial therapy, and electron beam or photon beam radiotherapies has shown efficacy in 90%–95% of cases (8). Fractionated treatments require daily sessions over several weeks, and although hypofractionated courses offer a convenient shorter schedule, risks of fibrosis, telangiectasia, and unsatisfactory cosmetic outcomes have been concerns although not always observed (11). In addition, many techniques require the patient to lie flat, which can be difficult for elderly patients (12).

Effective, nonsurgical treatments delivered quickly in an outpatient setting, particularly in a sitting or lying position, would represent a significant improvement in the standard of care. Rhenium skin cancer therapy (Rhenium SCT; OncoBeta International GmbH) is a novel epidermal radionuclide therapy for NMSC that offers promise in this space. Rhenium SCT utilizes the β-emitter radioisotope ^188^Re with a half-life of about 17 h. [^188^Re]resin is applied to an adhesive foil affixed to the target lesion, thus avoiding direct skin contact. The procedure is noninvasive and painless and can be administered without anesthesia over a short period of time in an outpatient setting.

Rhenium SCT has been successfully used in a variety of clinical trials over the past 15 y. Retrospective studies have shown response rates similar to those reported for either conventional or Mohs surgery, without scarring or the need for corrective or cosmetic repair (13–17). An early trial of 53 patients with histologically confirmed BCC or SCC received treatment with Rhenium SCT. A complete response (CR) was obtained within 3 mo in all patients, and no clinical relapses were observed (mean follow-up, 51 mo) (18). In a more recent trial of 43 patients, 29 with BCC and 14 with SCC, complete remission was reported for all lesions for which follow-up was available. No recurrences occurred during the follow-up period (mean, 288 d) (19).

The aim of the first prospective, multicenter, international, phase IV, single-arm study (EPIC-Skin) is to evaluate the safety and effectiveness of Rhenium SCT, along with important patient-reported outcomes, for the treatment of NMSC. Here, we present the interim study results of the first 106 lesions with 6-mo follow-up.

## MATERIALS AND METHODS

### Study Design and Objective

The EPIC-Skin trial (NCT05135052) is a prospective, multicenter, single-arm, open-label, phase IV study conducted at 7 sites worldwide: Australia (3 sites), South Africa (1 site), and Europe (Germany, Austria, and U.K., 1 site each). The objective is to assess clinic- and patient-reported outcomes of Rhenium SCT as a treatment for BCC and SCC. All patients will remain in the study for 24 mo from the time of their treatment with Rhenium SCT. Patients requiring further or alternative treatments for the target lesions during the trial period will leave the study. The 6-mo interim analysis described herein was scheduled for when 50% of the enrollment target recorded a 6-mo follow-up visit. The purpose of this analysis is to provide an initial indication of Rhenium SCT’s efficacy, safety, and associated patient-reported treatment comfort and QoL.

### Patient Population and Eligibility

The study was approved by the relevant institutional review boards at each participating site, and all patients provided written informed consent. Eligible patients (>18 y old) were those with up to 3 biopsy-proven BCC lesions or well-differentiated to moderately differentiated SCC lesions, up to 8 cm^2^ in size, with a depth of up to 3 mm, and clinically node-negative disease. Patients were required to have a Karnofsky performance status of at least 70%; provide informed consent; be determined ineligible for surgery because of tumor location, performance status, or other comorbidities deemed relevant by the treating clinician; or have declined surgery or fractionated radiation therapy. Patients were excluded from the study if they received prior treatment with surgery, radiation, or laser therapy for their target lesions. Tumors were excluded if they were affecting nerves or bony structures, if there were clinical concerns of metastatic disease, or if there was perineural or lymphovascular invasion affecting the medial canthus, eyelid margin (upper and lower), or vermilion lip. Patients were excluded if they had lupus or scleroderma, basal cell naevus syndrome, xeroderma, vitiligo, or albinism or were receiving ongoing systemic therapy for any malignancy or in the 4 wk before study entry. Patients who were pregnant, or for whom pregnancy could not be ruled out, were also excluded.

### Treatment

Rhenium SCT was administered on day 0 as a single treatment by application of [^188^Re]resin to an adhesive foil (Aerofilm; Aero Healthcare) affixed to the target lesion, such that radioactive material did not come into direct contact with the skin (19). The treatment area included the demarcated clinical disease plus a 5-mm margin to ensure adequate treatment of any subclinical disease. The treatment area was calculated manually by transcribing the lesion onto graphical measurement paper by the treating clinician. This measurement was validated by the supporting technician. The treatment time required to achieve the target dose at the defined depth was calculated on the basis of the activity (in MBq) of the [^188^Re]resin applied and the surface area treated. Treatment time required to achieve a 50-Gy dose to the base of the lesion (as measured by punch biopsy) was determined using VARSKIN 5 calculations that use Monte Carlo–based dose point kernels (20). The standardized 50-Gy dose was based on the optimal efficacy and safety profile of earlier studies (15). Validation of the delivered dose was verified previously using phantom-based experiments. The adhesive foil containing the [^188^Re]resin was removed after the predetermined treatment time necessary to deliver 50 Gy to the base of the lesion had elapsed. The facilities and personnel involved in treatment held appropriate local licenses to receive, handle, and dispose of radioactive material and practiced appropriate radiation protection and survey principles. All clinicians involved in the handling and administration of Rhenium SCT received standardized training, competency assessment, and certification by OncoBeta International GmbH after demonstrating proficiency in treating a minimum of 10 lesions across various anatomic zones.

### Assessments

A clinical trial smartphone application was utilized for this study to collect patient-reported outcomes, such as QoL and comfort-of-treatment questionnaires and monthly lesion photographs.

Tumor response was assessed according to a modified visual RECIST (21), with manual measurement of the longest diameter of the lesion at baseline and 6-mo follow-up by the treating clinician using the naked eye and a ruler or calipers. Responses were classified as follows: complete response (CR), with complete disappearance of target lesion; partial response (PR), with at least a 30% decrease in the largest diameter of the target lesion; progressive disease (PD), with at least a 20% increase in the largest diameter of the target lesion; and stable disease (SD), with neither a sufficient increase nor a sufficient decrease in the largest diameter to qualify as PR or PD.

QoL was assessed using Skin Cancer Index (22) questionnaires completed by patients at each time point. Treatment comfort was assessed using a questionnaire (Supplemental Table 1) completed by patients on day 14 follow-up. Cosmetic outcomes were assessed by both patient and clinician using the Cosmetic Outcome Visual Analog Scale, overall and by tumor type. Safety assessment included monitoring for adverse events (AEs) using Common Terminology Criteria for Adverse Events version 5.0, with an onset date before April 1, 2023, categorized by system organ class and preferred term of the Medical Dictionary for Regulatory Activities, by severity, and by relationship to Rhenium SCT. AEs of special interest assessed included radiation dermatitis, skin ulceration, alopecia, skin induration, hypo- or hyperpigmentation, and telangiectasia.

### Study Objectives

The primary outcome endpoint of the study is the proportion of lesions achieving a CR at 12 mo. For this interim analysis, tumor response was assessed at 6 mo. Secondary outcome measures are changes from baseline in QoL (adjusted mean change in the Skin Cancer Index score from baseline to 6-mo follow-up); treatment comfort (frequency and percentage of patients reporting each option on the questionnaire) at day 14; and cosmetic outcome (adjusted mean cosmetic outcome score, overall and by tumor type). Other outcome measures are safety, AEs, and toxicities.

### Sample Size

Calculation of the study sample size is based on the primary objective of the study to estimate 12-mo CR rate and to show noninferiority to historic values for CR rate after surgery or radiotherapy. The CR rate at 5 y is 91% for BCC (23) and 79% for SCC (24). Review of the published papers for brachytherapy shows that the split between BCC and SCC is 2:1 (19), 1.68:1 (15), and 2.1:1 (19). On the basis of previous studies, the CR rates for BCC and SCC were expected to be close to 100% after Rhenium SCT treatment. For simplicity, assuming 1 lesion per patient, a sample size of 120 patients is sufficient to provide at least 80% power to conclude noninferiority using a 1-sided α of 0.025.

### Ethics

The study is conducted in compliance with the protocol and ethical principles originating in or derived from the Declaration of Helsinki, institutional review board and independent ethics committees of each institution, informed-consent regulations, and the International Conference on Harmonization Good Clinical Practice guidelines (ISO 14155:2020) (25*,*^26^). In addition, all local legal and regulatory requirements will be followed.

## RESULTS

### Demographics

Between December 2021 and January 2023, 182 patients were enrolled into the study. Patient demographics are presented in Table 1. The median age was 72 y (range, 27–95 y). In total, 144 patients had BCC only (80.0%), 32 had SCC only (17.8%), and 4 had both BCC and SCC (2.2%). Two patients had no tumor information recorded and were screen failures. At the time of data cutoff (April 23, 2023), 81 patients (45.0%) had at least 1 postbaseline tumor assessment performed at 6 mo using modified visual RECIST, comprising a total of 106 tumors; 70 patients (38.9%) had at least 1 postbaseline Skin Cancer Index QoL assessment; and 149 patients (82.8%) had a day 14 treatment comfort assessment recorded.

**TABLE 1.** Patient Demographics

Characteristic	Overall (*n* = 182)*	BCC only (*n* = 144)	SCC only (*n* = 32)	Both BCC and SCC (*n* = 4)
Age (y)	70.6 ± 12.8	69.9 ± 13.0	73.5 ± 11.7	74.8 ± 8.3
Sex, *n* (%)				
Male	95 (53.7%)	73 (51.4%)	20 (64.5%)	2 (50.0%)
Female	82 (46.3%)	69 (48.6%)	11 (35.5%)	2 (50.0%)
Fitzpatrick skin type				
I	39 (23.6%)	32 (24.6%)	6 (19.4%)	1 (25.0%)
II	100 (60.6%)	79 (60.8%)	19 (61.3%)	2 (50.0%)
III	25 (15.2%)	19 (14.6%)	5 (16.1%)	1 (25.0%)
IV	0 (0.0%)	0 (0.0%)	0 (0.0%)	0 (0.0%)
V	0 (0.0%)	0 (0.0%)	0 (0.0%)	0 (0.0%)
VI	1 (0.6%)	0 (0.0%)	1 (3.2%)	0 (0.0%)
Tumor location, *n* (%)				
Nose	57 (23.4%)			
Other facial location	38 (15.6%)			
Other location	31 (12.7%)			
External ear	15 (6.1%)			
Back	14 (5.7%)			
Cheek	14 (5.7%)			
Forehead	14 (5.7%)			
Lower limbs	13 (5.3%)			
Scalp	11 (4.5%)			
Hand	9 (3.7%)			
Neck	8 (3.3%)			
Shoulder	8 (3.3%)			
Inner ear	4 (1.6%)			
Upper limbs	4 (1.6%)			
Knee	2 (0.8%)			
Foot	1 (0.4%)			
Perioral	1 (0.4%)			

* Two patients did not have type of skin cancer (BCC or SCC) recorded. These patients were both screen failures.

Continuous data are number and SD.

### RECIST Assessment at 6 Months

CRs were reported in 97.2% (95% CI, 92%–99.4%) (103/106) of tumors from the 81 patients assessed at 6 mo, with the remaining 2.8% (3/106) of tumors achieving PR (Table 2). Tumor-type–specific CRs were 97.6% (81/83) for BCC and 95.7% for SCC (22/23). There were 7 tumors included in the tumor assessment dataset for which no evaluation was provided.

**TABLE 2.** Modified Visual RECIST Categories for Intention to Treat Patient Tumors Evaluated at 6-Month Follow-up

Category	All tumors (*n* = 106)	BCC tumors (*n* = 83)	SCC tumors (*n* = 23)
CR	103 (97.2)	81 (97.6)	22 (95.7)
PR	3 (2.8)	2 (2.4)	1 (4.3)
PD	0 (0.0)	0 (0.0)	0 (0.0)
SD	0 (0.0)	0 (0.0)	0 (0.0)
Estimated CR rate at 6-mo follow-up	97.2% (92.0%–99.4%)	97.6% (91.6%–99.7%)	95.7% (78.1%–99.9%)

Patients with both BCC and SCC tumors are counted in both columns for these tumor types. Response data are number and percentage. Estimated CR rate is percentage and 95% CI.

### QoL

All subscales and total scores showed an increase in QoL, on average, from baseline to 6 mo (Table 3). For the total score, the average improvement was 7.88 points (on a 100-point scale); for the emotion subscale, the average improvement was 9.24 points; for the social subscale, the average improvement was 6.35 points; and for the appearance subscale, the average improvement was 8.06 points.

**TABLE 3.** Change from Baseline to 6-Month Follow-up in Skin Cancer Index Subscale and Total Scores

Change from baseline to 6 mo	Overall (*n* = 157)	BCC only (*n* = 123)	SCC only (*n* = 30)	Both BCC and SCC (*n* = 4)
Total SCI score	7.88 (14.0)	9.29 (14.79)	2.79 (11.9)	7.94 (1.1)
Emotion subscale	9.24 (16.4)	9.99 (17.5)	4.90 (12.5)	19.64 (2.5)
Social subscale	6.35 (12.0)	7.42 (12.4)	3.46 (11.4)	0.00 (0.0)
Appearance subscale, *n*	8.06 (22.6)	10.46 (22.9)	0.00 (22.0)	4.17 (5.9)

SCI = Skin Cancer Index.

Data are mean and SD.

**FIGURE 1.**
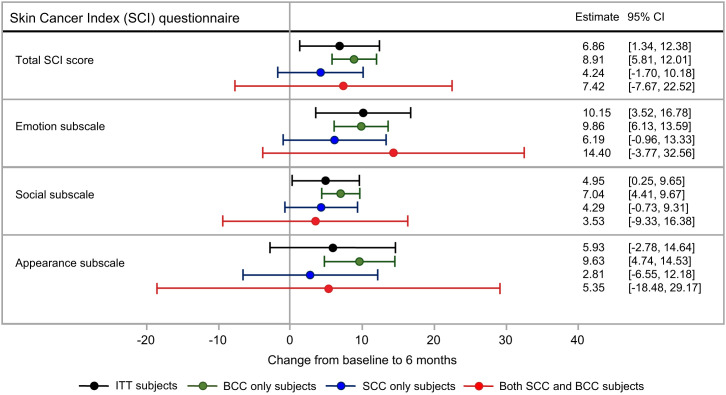
Estimated change from baseline to 6-mo follow-up for Skin Cancer Index subscales, adjusted for baseline subscale scores. ITT = intention to treat.

Additional analysis was undertaken on the change from baseline to 6 mo via a repeated-measures model. The baseline value for each subject was included as a covariate in the model, as was age (under vs. over 70 y of age). The results from the fitted model are generally similar to the summary statistics (Fig. 1).

### Treatment Comfort

In total, 126 patients completed the treatment-related questions (Supplemental Table 1), and of those, 100% reported no treatment pain or discomfort.

### Safety

There were 40 treatment-emergent AEs reported in 29 patients (15.9% of patients with at least 1 AE) (Table 4). AEs were grade 1 (25 events) or grade 2 (13 events); 2 events did not have a grading provided at this time. A relationship to Rhenium SCT was noted as probable for 22 events, possible for 7 events, unrelated for 6 events, and missing for 5 events.

**TABLE 4.** Treatment-Emergent AEs

Characteristic	Data (total *n* = 182)
Any adverse events	29 (15.9)
Serious events	1 (0.5)
Events leading to withdrawal	0 (0.0)
Severity	
Mild	19 (10.4)
Moderate	9 (4.9)
Severe	0 (0.0)
Missing	2 (1.1)
Relationship to Rhenium SCT	
Unrelated	5 (2.7)
Possibly related	7 (3.8)
Probably related	17 (9.3)
Missing	4 (2.2)

Data are number and percentage.

Treatment AEs (defined as AEs that were possibly, probably, or definitely related to Rhenium SCT) were reported in 24 patients (29 treatment-related events). AEs were mostly grade 1 or grade 2. The most frequently reported events by the Medical Dictionary for Regulatory Activities’ preferred term were pain (7 events in 7 patients), wound infection (3 events in 3 patients), swelling of the face (2 events in 2 patients), skin pain (2 events in 2 patients), skin infection (2 events in 2 patients), urticaria (2 events in 2 patients), and wound complication (2 events in 2 patients) (Supplemental Table 2). Additionally, 1 grade 3 AE of nonhealing for a duration greater than 6 mo was reported; this has since resolved. There were no AEs that led to study withdrawal.

## DISCUSSION

There are many treatments for NMSC, including surgery, cryotherapy, curettage, topicals, brachytherapy, or conventional external beam radiotherapy. The most appropriate option depends on clinical, technical, and logistic factors and, importantly, patient wishes. This interim analysis of the largest global prospective study (EPIC-Skin), to our knowledge, of Rhenium SCT for BCC and SCC is a critical efficacy and safety evaluation of the noninvasive single-session treatment. These data are essential to modify treatment paradigms and to improve access to Rhenium SCT for suitable patients.

This 6-mo analysis showed that Rhenium SCT was effective and well tolerated with an acceptable treatment safety profile and was associated with improved QoL across all Skin Cancer Index subscales. AEs reported were mostly grade 1 or grade 2 and did not result in study withdrawal. Of the 106 tumors from 81 patients assessed at 6 mo, 97.2% achieved a CR, with a PR elicited in the remaining tumors. Encouragingly, CR rates for BCC and SCC are similar at 97.6% and 95.7%, respectively, where treatments for SCC traditionally yield lower efficacy rates. Long-term follow-up is required for this cohort, however, to assess the duration of response. This is consistent with outcomes from a systemic review of NMSC treated with fractionated high-dose-rate brachytherapy, yielding median local control rates of 97% and good or excellent cosmetic outcomes in 95% of cases (27). These data indicate that appropriately targeted radiation-based treatment can elicit CR rates similar to those with surgery.

Rhenium SCT offers multiple practical and patient-specific benefits for relevant indications. These include reduced procedural and postoperative pain, reduced disfiguration and scarring, improved health-related QoL, and improved anatomic functionality (28). Rhenium SCT offers a single-session treatment with efficacy comparable to that of traditional radiotherapy modalities but with greater planning and treatment convenience.

Further clinical data from this study, including the 12-mo tumor response primary endpoint, are required to demonstrate long-term efficacy, safety, and patient-reported outcome measures.

Limitations of the analysis include that it is an interim analysis. Longer-term follow-up is required to reach all defined endpoints. For the analysis of change from baseline in Skin Cancer Index subscales, only 1 follow-up measure was able to be included per patient, and therefore, the model is essentially a linear regression model with no repeated measures at this point. Further patient reporting points as the study progresses will be valuable.

## CONCLUSION

Rhenium SCT epidermal radionuclide therapy offers an important nonsurgical modality in the treatment armamentarium available to indicated patients with BCC and SCC. It is a noninvasive and painless procedure, usually delivered as a single outpatient session, without the need for anesthesia. It benefits patients who are concerned about cosmetic or functional outcomes from surgery, who are not ideal surgical candidates, and who may be unsuitable for a fractional course of conventional radiotherapy.

## DISCLOSURE

This study was funded by OncoBeta Therapeutics Australia. Siddhartha Baxi, Saima Vohra, and Gerhard Dahlhoff work as medical consultants for OncoBeta. Angela Hong has received compensation from OncoBeta for participation in Advisory Boards. Martin Heuschkel has received research funding from OncoBeta. No other potential conflict of interest relevant to this work was reported.

## Notes

KEY POINTS **QUESTION:** Does Rhenium SCT for NMSC improve the clinical efficacy and patient-reported outcomes? **PERTINENT FINDINGS:** NMSC lesions treated with a single session of Rhenium SCT had a CR rate of 97.2%, and patients reported significant improvements in QoL. **IMPLICATIONS FOR PATIENT CARE:** Rhenium SCT is an effective and noninvasive treatment for NMSC in patients who are unsuitable for, or decline, surgery.
